# Bi-level graph attention paradigm with differential strategy integration for heterogeneous multi-agent reinforcement learning

**DOI:** 10.1038/s41598-026-41722-w

**Published:** 2026-03-05

**Authors:** Yun Li, Zhimin Zhang, Jiao Wang

**Affiliations:** 1 School of Information Science and Engineering, Northeastern University, Shenyang, 110001 China; 2 Institute of Information Technology, Luoyang Normal University, Luoyang, 471000 China; 3 Henan Zhongyuan Big Data Research Institute Co., Ltd, Luoyang, 471000 China

**Keywords:** Heterogeneous games, Multi-agent reinforcement learning, Graph attention network, Strategy integration, Mathematics and computing, Physics

## Abstract

Collaboration among heterogeneous agents is crucial for addressing complex real-world tasks that require leveraging diverse capabilities. In such systems, increasing agent numbers amplify the challenges of communication and coordinated decision-making, in addition to the inherent heterogeneity of the agents. To address these issues, we propose the Bi-level Graph Attention Paradigm (Bi-GAP) with differential strategy integration, a novel policy-based group learning framework designed for heterogeneous Multi-Agent Systems (MAS) in both discrete and continuous domains. Bi-GAP employs a bi-level graph attention architecture to model intricate interaction patterns among isomorphic agents within groups and across heterogeneous groups. This hierarchical representation enables flexible and selective communication, reduces unnecessary message exchange, and improves the robustness of the MAS under interference. Furthermore, the framework integrates multi-perspective strategies, allowing each member-agent to incorporate global guidance from its designated guide-agent while still performing fine-grained local reasoning. This mechanism balances macro-level coordination with micro-level adaptability. We evaluate Bi-GAP on heterogeneous StarCraft II micromanagement tasks and Multi-Agent Particle Environment Predator–Prey scenarios. The results show that Bi-GAP consistently outperforms recent state-of-the-art MARL baselines across both discrete and continuous settings.

## Introduction

Multi-agent systems (MAS) are effective for addressing complex problems beyond the capability of a single agent, such as robotics^1^, autonomous vehicles^2,3^, games^4^, and unmanned aerial vehicles^5^. Due to the complex structures and behaviors of those systems, the optimal strategy of an individual agent may not be suitable for the entire system, emphasizing the need for considering interactions and cooperation among multiple agents. Therefore, it is crucial to enable agents to continuously learn from their experiences, capture the non-linear relationships among them, and adapt to the complexity and dynamics of the environment and other agents. Multi-agent reinforcement learning (MARL), a technique of deep reinforcement learning (DRL)^6^ designed for MAS, has shown great promise in learning coordinated behaviors among multiple agents, including those with heterogeneous properties.

MARL encounters significant challenges in solving complex multi-agent games due to partial observability, scalability limitation and non-stability. Pure centralized^7^ and pure decentralized^8^ learning have been proposed to address these challenges but have limitations. They adopt complete and partial observation, respectively, with the former exhibiting good stability but suffering from scalability issues, and the latter presenting the opposite trade-off. To achieve a balance, researchers have explored integrating the two methods through the Centralized Training and Decentralized Execution (CTDE) framework^9,10^ and the Master-Slave Architecture^11,12^ in a complementary manner. The two frameworks both leverage global and local information, but vary in optimizing strategies of agents. In the CTDE paradigm, the critic provides value, akin to judges scoring games, while the master agent in the Master-Slave Architecture offers explicit guidance for policy optimization, similar to coaches guiding game strategies. This kind of coaching guidance has a more direct effect on policy optimization and is more conducive to agent learning. However, as the number of agents and their heterogeneity increase, communication overhead, non-stationarity, partial observability, and decision-coordination complexity rise significantly, making it difficult for conventional CTDE frameworks to handle these challenges. At the same time, the reliance of the master-slave architecture on a single master agent renders it insufficiently robust and inadequate for multi-agent systems with a growing number of heterogeneous agents.

In reality, sophisticated tasks often require many agents with distinct capabilities to collaborate and leverage their strengths. For instance, a project involving multiple organizational departments—such as marketing, sales, technology, and finance—typically includes several members in each department, with the importance and responsibilities of each department varying across different project phases. Managers may serve as key coordinators, providing guidance for decision optimization, while other members have limited involvement in information exchange. Attention-based group communication methods^13–15^, have demonstrated promising results in mitigating the challenges introduced by increasing agent numbers, offering valuable insights for designing scalable communication mechanisms. However, existing MARL approaches for heterogeneous environments^16–18^ primarily rely on value function decomposition and optimize agent policies in a generic manner. These methods often overlook the heterogeneity and distinct characteristics of individual agents, which can severely limit the performance of multi-agent systems. Therefore, it is crucial to design communication channels and decision-optimization mechanisms that not only accommodate the increasing number of agents but also effectively exploit the differences among heterogeneous agents, enabling coordinated and efficient collaboration across multiple task phases.

We herein propose a novel policy-based group learning technique called the Bi-level Graph Attention Paradigm (Bi-GAP) for both discrete and continuous heterogeneous MAS. In our approach, agents are first grouped based on their types, with an extra guide-agent assigned to each group, and its members referred to as member-agents. Subsequently, a Bi-level Graph Attention Network is presented to dynamically interact information among diverse agents. The isomorphic level describes the importance distribution of isomorphic member-agents, while the heterogeneous level models relationships among heterogeneous groups through guide-agents. Finally, to optimize the actions of the agents, we develop a strategy similarity based strategy integration technique. Member-agents execute actions based on the integrated strategy, which incorporates their individual reasoning and exclusive guidance from the corresponding guide-agent intelligently. The key contributions of our approach can be summarized as follows:

- We establish a bi-level graph attention–based group learning framework that enables real-time interaction modeling among homogeneous and heterogeneous agents under partial observability, while reducing redundant communication to enhance system security by limiting information exposure, mitigating attack surfaces, and improving robustness against false information injection.
- We propose an adaptive strategy integration method that combines each member-agent’s local policy with its guide-agent’s global policy. The guide-agent provides macro-level correction when deviations are large, while similar strategies allow member-agents to focus on fine-grained decision-making.

## Related work

In the past decade, reinforcement learning (RL)^19^ has successfully tackled complex sequential decision-making problems. Recently, RL has been extended to continuous state spaces using neural networks in multi-agent reinforcement learning (MARL), enabling efficient feature representation for various applications like robotics^20^ and games^21^. However, MARL faces challenges in making appropriate decisions due to incomplete observable information, non-stationary environments resulting from complex agent interactions, dynamic characteristics, and exponential growth of the action space with more agents. Purely centralized^7^ and purely decentralized^22,23^ learning approaches alone cannot address these challenges. To overcome this, researchers have explored two complementary approaches: the CTDE framework and the master-slave architecture.

### CTDE framework

The CTDE framework integrates both centralized and decentralized learning by training centralized critics with global information and having decentralized actors perform actions on the environment with local information. This approach effectively addresses the curse of dimensionality in centralized learning while improving convergence in decentralized learning. Classic algorithms based on the CTDE framework, such as MADDPG^9^ and COMA^10^, have been proposed to handle continuous and discrete multi-agent environments, respectively. However, the scalability of these approaches in MARL scenarios is hindered as the number of agents increases, due to the need for information exchange among all agents for centralized critics.

Attention-based group communication attempts to alleviate the constraints associated with an increasing number of agents, e.g., G2ANet^24^, HAMA^13^, DGN^14^, AHAC^15^. For instance, G2ANet establishes communication by a two-stage attention network, where hard-attention and soft-attention are used to construct communication group and learn important weight within group. However, hard attention simplifies communication by completely discarding some elements, which may lead to incomplete information. HAMA maintains continuous inter-agent and inter-group communication, which results in high communication overhead and poses significant practical implementation challenges. Therefore, it is crucial to design a communication method for heterogeneous multi-agent systems that maintains accuracy while minimizing redundant exchanges as agent numbers grow.

Furthermore, the critics in CTDE behave similarly to judges in games, only providing values to the actors without offering explicit strategic guidance. However, if a role is able to give explicit strategic guidance, akin to that of a coach in a game, it can greatly facilitate the convergence of strategies. This is precisely what we introduce next as the master-slave architecture.

### Master-slave architecture

The master-slave architecture has been extensively studied in various MARL scenarios^11,12^. In this architecture, the master agent assumes the role of a coach and provides explicit guidance for policy learning, combining centralized and decentralized control explicitly. For instance, in MS-MARL^11^, the slave agents act by combining the reasoning of the master agent and their own individual thinking. However, the lack of communication among the slave agents limits potential cooperation. Additionally, the equal weighting and combination of the policy guidance from the master agent and the individual policy reasoning of the slave agents restrict the accurate representation of the composed action. Therefore, a more reasonable decision integration technique is crucial to optimize the strategy.

More importantly, regardless of CTDE framework or master-slave architecture, the above approaches solely explor the coordination among isomorphic agents, and neglect the variations in attribution among heterogeneous agents. To address this concern, algorithms based on value decomposition, such as Qmix^16^, ThGC^17^, and LFMCO^18^, have been proposed. These algorithms have shown promising performance in heterogeneous multi-agent cooperation. However, the monotonic relationship between joint values and individual values, as well as its inadequacy to explore the differences among heterogeneous agents, prevent agents from achieving the optimal joint value function. Moreover, the extensive value decomposition calculations of Qmix and LFMCO constrain their ability to solve large-scale tasks. The structure of group learning in THGC guarantees its application in large-scale missions, which is worth learning. Furthermore, it is noteworthy to revive the LFMCO, shares similar idea with the master-slave architecture and employs an leader-following paradigm. Both of them only employ a single master agent or leader to direct other agents, but this guidance is not precise in heterogeneous environments. Thus, it is more justifiable to assign distinct leaders for various attributes of agents to achieve more accurate policy representation.

## Methodology

In this section, a novel group learning algorithm is presented for medium-scale heterogeneous MAS. Firstly, a detailed description of the Bi-level Graph Attention Paradigm (Bi-GAP) with differential strategy integration is provided. Next, the theory of policy updating is discussed.

### Bi-level graph attention paradigm with differential strategy integration

**Fig. 1:**
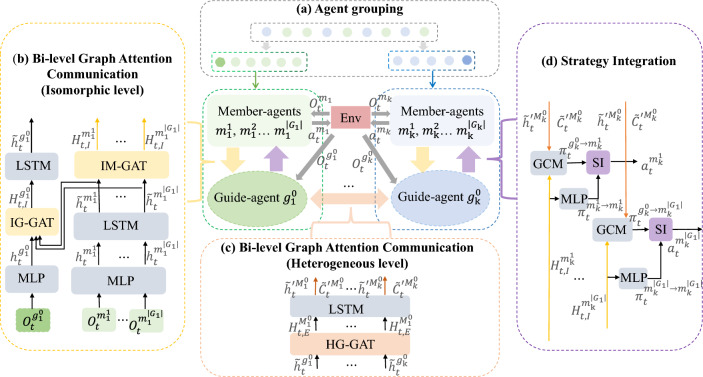
Bi-GAP framework: Bi-GAP consists of (**a**) agent grouping, which groups agents into distinct groups according to their types, (**b**) and (**c**) isomorphic and heterogeneous levels in Bi-level graph attention communication, depicting the interaction between isomorphic agents and heterogeneous groups respectively, and (**d**) strategy integration across multiple perspectives to optimize the decisions of agents.

The primary objective of Bi-GAP is to foster cooperation among agents in heterogeneous environments through communication improvement and decision optimization. The framework of the Bi-GAP is illustrated in Fig. 1. Notably, building upon the agent grouping, an extra virtual guide-agent is allocated to each group, and agents contained within each group are designated as member-agents. Member-agents communicate at an isomorphic level, whereas guide-agents engage in inter-group communication at a heterogeneous level after consolidating information within the group. Guide-agents solely provide policy guidance to their member-agents without taking actions. To optimize the actions of member-agents, a new technique is designed to integrate their local strategies and global strategies from the guide-agent. A comprehensive description of the Bi-GAP follows.

#### Agent grouping

Natural biological systems often exhibit division of labor and coordination among different types of organisms, such as bee colonies. Taking inspiration from these biological systems, we employ a similar approach by clustering our agents into separate groups based on their types. An extra virtual guide-agent is assigned for each group, within which all the isomorphic entity agents are defined as member-agents. Note that, both entity member-agents and virtual guide-agents are capable of reasoning. However, the former engage in actual cooperation, but the later does not, and only provides individual guidance for their member-agents. To depict the relationship among agents, an undirected graph $G=(N,E)$ is introduced, where the node set *N* represents the agents, and the edge set *E* signifies the interaction between two connected agents. For a system with *K* types agents, a sub-graph $G_k$ is established for group *k*, resulting in a graph *G* that comprises *K* sub-graphs. Specifically, $N=\sum _{k=1}^{K} \left| G_{k} \right|$, consisting of $N-K$ entity member-agents and *K* virtual guide-agents. As an example, a group *k* is referred to as:

1 $$\begin{aligned} G_{k}=\left\{ g_{k}^{0},m_{k}^{1},m_{k}^{2},\cdots ,m_{k}^{\left| G_{k} \right| } \right\} . \end{aligned}$$

where $g_{k}^{0}$ and $m_{k}^{j}$ denote the guide-agent and member-agent within group *k*, respectively. $G_{k}^{g}=\left\{ g_{1}^{0}, g_{2}^{0},\cdots , g_{k}^{0} \right\}$ represents the set of all the guide-agents. The isomorphic member-agents are connected to each other within their own group, whereas only guide-agents are connected across different groups.

#### Bi-level graph attention communication

In heterogeneous multi-agent group learning, effective interaction between isomorphic and heterogeneous agents is crucial. It is essential to dynamically capture and describe the interactive relationships among isomorphic agents and heterogeneous groups in real-time. In this part, a Bi-level graph attention network is introduced to promote information interaction, ensuring efficient communication while avoiding redundancy.

Before presenting our communication model, let’s describe the information processing required upfront. As exhibited in Fig. 1a, agents are divided into *K* groups according to their types. At the time step *t*, the guide-agent $g_{k}^{0}$ of the group *k* holds the global observation $o_{t}^{g_{k}^{0} }$. The member-agents within the group have similar observations $o_{t}^{m_{k}^{i} }$. A Multi-Layer Perceptron ( MLP), a type of feed forward artificial neural network, is used to understand the environment. The $o_{t}^{g_{k}^{0} }$ and $o_{t}^{m_{k}^{i} }$ are encoded as $h_{t}^{g_{k}^{0} }$ and $h_{t}^{m_{k}^{i} }$, respectively. Note that, agents of the same type exhibit more consistent cognition about the environments, thus we consider that the isomorphic agents share MLP networks. Next, the isomorphic-agent communication and heterogeneous-agent communication will be demonstrated respectively.

(1) Isomorphic level graph attention communication

For the member-agents within each group, just communication in isomorphic level is considered, which happens after extracting features. Specifically, after the environment is cognized at time step *t*, an LSTM layer is employed to extract the feature and obtain $\tilde{h}{t}^{m{k}^{i}}$,

2 $$\begin{aligned} \tilde{h}_{t}^{m_{k}^{i} } =LSTM({h}_{t}^{m_{k}^{i} }). \end{aligned}$$

where ${h}_{t}^{m_{k}^{i} }$ is the environment cognition of member-agent $m_{k}^{i}$ in group *k*, which is encoded from observation $o_{t}^{m_{k}^{i} }$.

Then, the Isomorphic-member GAT (Im-GAT) is used by member-agents for isomorphic-level communication. The attention score between member-agents $m_{k}^{i}$ and $m_{k}^{j}$ is calculated by:

3 $$\begin{aligned} & e_{m_{k}^{i} m_{k}^{j}}=att\left( \tilde{h}_{t}^{m_{k}^{i} }, \tilde{h}_{t}^{m_{k}^{j} }\right) . \end{aligned}$$

4 $$\begin{aligned} & \alpha _{m_{k}^{i} m_{k}^{j}}=\operatorname {softmax}_{m_{k}^{j}}\left( e_{m_{k}^{i} m_{k}^{j}}\right) =\frac{\exp \left( e_{m_{k}^{i} m_{k}^{j}}\right) }{\sum _{m_{k}^{n} \in \mathcal {G}_{k}} \exp \left( e_{m_{k}^{i} m_{k}^{n}}\right) }. \end{aligned}$$

where the $e_{m_{k}^{i} m_{k}^{j}}$ represents the correlation between $m_{k}^{i}$ and $m_{k}^{j}$. $\alpha _{m_{k}^{i} m_{k}^{j}}$ expresses the attention coefficient which is obtained by normalization function softmax. Based on above attention coefficient, the features of member-agents in the same group were aggregate with weight:

5 $$\begin{aligned} H_{t,I}^{m_{k}^{i} } =\sigma \left( \sum _{m_{k}^{j} \in \mathcal {G}_{k} } \alpha _{m_{k}^{i} m_{k}^{j}} \tilde{h}_{t}^{m_{k}^{j} }\right) . \end{aligned}$$

where $H_{t,I}^{m_{k}^{i} }$ is the output of I-GAT, the new feature of $m_{k}^{i}$, which incorporates the information of all other member-agents in the group.

When the above member-agents carry out isomorphic communication, the guide-agent performs isomorphic communication by merging the information provided by its member-agents in preparation for subsequent heterogeneous communication. Varying from member-agents, guide-agents first integrate information and then extract features. The Isomorphic-guide GAT (Ig-GAT) is adopted by guide-agent to communicate in isomorphic level, the specific steps are as follows:

6 $$\begin{aligned} & e_{g_{k}^{0} m_{k}^{j}}=att\left( {h}_{t}^{g_{k}^{0}}, {h}_{t}^{m_{k}^{j} }\right) . \end{aligned}$$

7 $$\begin{aligned} & \alpha _{g_{k}^{0} m_{k}^{j}} =\operatorname {softmax}_{m_{k}^{j}}\left( e_{g_{k}^{0} m_{k}^{j}}\right) = \frac{\exp \left( e_{g_{k}^{0} m_{k}^{j}}\right) }{\exp \left( e_{g_{k}^{0} g_{k}^{0}}\right) + \sum _{m_{k}^{n} \in \mathcal {G}_{k}} \exp \left( e_{g_{k}^{0} m_{k}^{n}}\right) }. \end{aligned}$$

The correlation and attention coefficient between each guide-agent and its member-agents are represented by $e_{g_{k}^{0} m_{k}^{j}}$ and $\alpha _{g_{k}^{0} m_{k}^{j}}$ respectively. The information representations of all agents in the group *k*, including member and guide agents, are aggregated according to the attention coefficient $\alpha _{g_{k}^{0} m_{k}^{j}}$ by guide-agents:

8 $$\begin{aligned} H_{t,I}^{g_{k}^{0} } =\sigma \left( \alpha _{g_{k}^{0} g_{k}^{0}} {h}_{t}^{g_{k}^{0} } +\sum _{m_{k}^{j} \in \mathcal {G}_{k} } \alpha _{g_{k}^{0} m_{k}^{j}} {h}_{t}^{m_{k}^{j} }\right) . \end{aligned}$$

The features of $H_{t,I}^{g_{k}^{0} }$ are extracted by LSTM to ontain $\tilde{h}_{t}^{g_{k}^{0} }$:

9 $$\begin{aligned} \tilde{h}_{t}^{g_{k}^{0} }=LSTM(H_{t,I}^{g_{k}^{0} }). \end{aligned}$$

(2) Heterogeneous level graph attention communication

Communication in heterogeneous level is achieved by the guide-agents completely. The Heterogeneous-guide GAT (Hg-GAT) is introduced to learn relationships among heterogeneous groups. Details are as follows.

10 $$\begin{aligned} & e_{g_{i}^{0} g_{j}^{0}}=att\left( \tilde{h}_{t}^{g_{i}^{0} }, \tilde{h}_{t}^{g_{j}^{0} }\right) . \end{aligned}$$

11 $$\begin{aligned} & \alpha _{g_{i}^{0} g_{j}^{0}}=\operatorname {softmax}_{g_{j}^{0}}\left( e_{g_{i}^{0} g_{j}^{0}}\right) =\frac{\exp \left( e_{g_{i}^{0} g_{j}^{0}}\right) }{\sum _{g_{n}^{0} \in \mathcal {G}_{k}^{g}} \exp \left( e_{g_{i}^{0} g_{n}^{0}}\right) }. \end{aligned}$$

12 $$\begin{aligned} & H_{t,H}^{g_{i}^{0} }=\sigma \left( \sum _{g_{j}^{0} \in \mathcal {G}_{k}^{g} } \alpha _{g_{i}^{0} g_{j}^{0}} \tilde{h}_{t}^{g_{j}^{0} }\right) . \end{aligned}$$

The current discussion omits an explanation of functions that repeat described earlier. Here, $G_{k}^{g}$ denotes the collection of all guide-agents within the system. The correlation coefficient and attention coefficient are respectively represented by $e_{g_{i}^{0} g_{j}^{0}}$ and $\alpha _{g_{i}^{0} g_{j}^{0}}$, both between pairs of guide-agents and between groups. Additionally, $H_{t,E}^{g_{i}^{0}}$ signifies the updated feature set of guide-agent $g_{i}^{0}$, which integrates features of other heterogeneous agents.

The feature from $H_{t,E}^{g_{i}^{0} }$ is extracted by another LSTM:

13 $$\begin{aligned} \tilde{h}_{t}^{\prime g_{k}^{0} }, \tilde{c}_{t}^{\prime g_{k}^{0} }=LSTM(H_{t,H}^{g_{k}^{0} }, \tilde{h}_{t-1}^{\prime g_{k}^{0} } \tilde{c}_{t-1}^{\prime g_{k}^{0} }). \end{aligned}$$

where $\tilde{h}_{t}^{\prime g_{k}^{0} }$ and $\tilde{c}_{t}^{\prime g_{k}^{0} }$ are the hidden and cell states of the LSTM for inter-group feature extraction at time-step *t*, $\tilde{h}_{t-1}^{\prime g_{k}^{0} }$ and $\tilde{c}_{t-1}^{\prime g_{k}^{0} }$ are the similar ones at time-step $t-1$.

#### Strategy integration

For heterogeneous multi-agent cooperative tasks, apart from efficient communication, decision optimization is also of utmost importance. Inspired by MS-MARL^11^, the guide-agent is introduced for each group to provide precise guidance for the strategies of member-agents. Rather than simply averaging the policies from the master agent and slave agent in MS-MARL, an entropy weight is employed to integrate the strategies of the guide-agent and its member-agents. This approach strikes a well balance of maintaining the member-agent reasoning while simultaneously augmenting it with the guide-agent thinking.

The $H_{t,I}^{m_{k}^{i} }$ is the new features of member-agent $m_{k}^{i}$ acquired by the isomorphic-agent communication. The GCM, a gated composition module, is employed to obtain the unique guidance from the guide-agent. The MLP network $f\left( \cdot \right)$ is adopted to output the reasoning of member-agent. The details as follow:

14 $$\begin{aligned} & GCM(\tilde{h}_{t}^{\prime g_{k}^{0} }, \tilde{c}_{t}^{\prime g_{k}^{0} },H_{t,I}^{m_{k}^{i} })={\left\{ \begin{array}{ll} & \pi _{t}^{g_{k}^{0}\rightarrow m_{k}^{i}} \text { if action is discrete } \\ & \mu _{t}^{g_{k}^{0}\rightarrow m_{k}^{i}} \text { if action is continuous} \end{array}\right. }. \end{aligned}$$

15 $$\begin{aligned} & f(H_{t,I}^{m_{k}^{i} })={\left\{ \begin{array}{ll} & \pi _{t}^{m_{k}^{i}\rightarrow m_{k}^{i}} \text { if action is discrete } \\ & \mu _{t}^{m_{k}^{i}\rightarrow m_{k}^{i}} \text {if action is continuous} \end{array}\right. }. \end{aligned}$$

where the $\tilde{h}_{t}^{\prime g_{k}^{0} }$ and $\tilde{c}_{t}^{\prime g_{k}^{0} }$ are the parameters of GCM at time-step *t*, which is migrated from heterogeneous-agent feature extraction network LSTM in time-step *t*. Additionally, when the action is discrete, the thinking of guide-agent and member-agent is represented by the discrete probability distribution $\pi _{t}^{g_{k}^{0}\rightarrow m_{k}^{i}}$ and $\pi _{t}^{m_{k}^{i}\rightarrow m_{k}^{i}}$, respectively; In case the action is continuous, they are expressed by the mean of a Gaussian policy distribution, denoted as $\mu _{t}^{g_{k}^{0}\rightarrow m_{k}^{i}}$ and $\mu _{t}^{m_{k}^{i}\rightarrow m_{k}^{i}}$, respectively.

The $CEH(\pi _{t}^{g_{k}^{0}\rightarrow m_{k}^{i}},\pi _{t}^{m_{k}^{i}\rightarrow m_{k}^{i}})$ denotes the cross-entropy between the guidance from guide-agent and the thinking of member-agent. It is used to fuse the strategies and derive the probability distribution $\pi _{t}^{m_{k}^{i}}$ for discrete actions, as well as the mean $\mu _{t}^{m_{k}^{i}}$ for continuous actions. Subsequently, the action $a_{t}^{m_{k}^{i}}$ of member-agent $m_{k}^{i}$ is generated by sampling from either the softmax policy or Gaussian policy.

16 $$\begin{aligned} & \begin{aligned}&CEH(\pi _{t}^{g_{k}^{0}\rightarrow m_{k}^{i}},\pi _{t}^{m_{k}^{i}\rightarrow m_{k}^{i}}) \\& \quad ={\left\{ \begin{array}{ll} & -\sum _{a\in A}\pi _{t}^{g_{k}^{0}\rightarrow m_{k}^{i}}(a)\cdot \log {\pi _{t}^{m_{k}^{i}\rightarrow m_{k}^{i}}(a)} \text { if action is discrete} \\ & -\int \pi _{t}^{g_{k}^{0}\rightarrow m_{k}^{i}}(a) \cdot \log {\pi _{t}^{m_{k}^{i}\rightarrow m_{k}^{i}}(a)} \textrm{d}a \text { if action is continuous} \end{array}\right. }. \end{aligned} \end{aligned}$$

17 $$\begin{aligned} & \begin{aligned} \pi _{t}^{m_{k}^{i}} =CEH(\pi _{t}^{g_{k}^{0}\rightarrow m_{k}^{i}},\pi _{t}^{m_{k}^{i}\rightarrow m_{k}^{i}})\times \pi _{t}^{g_{k}^{0}\rightarrow m_{k}^{i}} +(1-CEH(\pi _{t}^{g_{k}^{0}\rightarrow m_{k}^{i}},\pi _{t}^{m_{k}^{i}\rightarrow m_{k}^{i}}))\times \pi _{t}^{m_{k}^{i}\rightarrow m_{k}^{i}}. \end{aligned} \end{aligned}$$

18 $$\begin{aligned} & \begin{aligned} \mu _{t}^{m_{k}^{i}} =CEH(\pi _{t}^{g_{k}^{0}\rightarrow m_{k}^{i}},\pi _{t}^{m_{k}^{i}\rightarrow m_{k}^{i}})\times \mu _{t}^{g_{k}^{0}\rightarrow m_{k}^{i}} +(1-CEH(\pi _{t}^{g_{k}^{0}\rightarrow m_{k}^{i}},\pi _{t}^{m_{k}^{i}\rightarrow m_{k}^{i}}))\times \mu _{t}^{m_{k}^{i}\rightarrow m_{k}^{i}}. \end{aligned} \end{aligned}$$

19 $$\begin{aligned} & a_{t}^{m_{k}^{i}}={\left\{ \begin{array}{ll} & softmax(\pi _{t}^{m_{k}^{i}}) \text { if action is discrete } \\ & \mu _{t}^{m_{k}^{i}}+\sigma \cdot N(0,1) \text { if action is continuous } \end{array}\right. }. \end{aligned}$$

The member-agent possesses more detailed information. When its strategy closely aligns with the guide-agent, the cross-entropy is low. To optimize the decision of the member-agent, priority is given to its thought processes, maintaining micro-level cognition. Conversely, if there is a significant difference between their strategies, resulting in high cross-entropy, the reasoning of the guide-agent takes precedence, leveraging its broader macro-level information. Thus, the decision of the member-agent can be optimized from a macro perspective.

### Policy updating

In this paper, the strategy is learned in an end-to-end manner using the Proximal Policy Optimization (PPO) algorithm, selected for its high sample efficiency and effective learning performance. A value network is incorporated to facilitate policy optimization by providing stable value estimates, and sample efficiency is further enhanced through multiple gradient-based updates using importance-sampling corrections. To maximize the action advantage of each member-agent, the strategy network is trained with a loss function that integrates policy improvement, variance reduction, entropy-driven exploration, and group-aware optimization. This loss function, presented in Equation (20), forms the foundation of stable and efficient multi-agent learning within the proposed Bi-GAP framework.

20 $$\begin{aligned} \mathcal {L}(\theta )= & \left[ \frac{1}{N_{batch}} \sum _{i=1}^{B} \sum _{k=1}^{K} \sum _{j=1}^{ \left| G_{k} \right| -1 } \operatorname {min}\left( r_{\theta , i,t }^{m_{k}^{j} } A_{i,t}^{m_{k}^{j}}, \operatorname {clip}\left( r_{\theta , i,t }^{m_{k}^{j} }, 1-\epsilon , 1+\epsilon \right) A_{i,t}^{m_{k}^{j}} \right) \right] \nonumber \\ & +\sigma \frac{1}{N_{batch}} \sum _{i=1}^{B} \sum _{k=1}^{K} \sum _{j=1}^{ \left| G_{k} \right| -1 } S\left[ \pi _{\theta }\left( s_{i,t}^{(m_{k}^{j})}\right) \right] \end{aligned}$$

21 $$\begin{aligned} A_{i,t}^{m_{k}^{j}}= & \delta _{i,t}^{m_{k}^{j}}+(\gamma \lambda ) \delta _{i,t+1}^{m_{k}^{j}}+\cdots +\cdots +(\gamma \lambda )^{T-t+1} \delta _{i,T-1}^{m_{k}^{j}}. \end{aligned}$$

22 $$\begin{aligned} r_{\theta , i,t }^{m_{k}^{j} }= & \frac{\pi _{\theta }\left( a_{i,t}^{m_{k}^{j}} \mid s_{i,t}^{m_{k}^{j}}\right) }{\pi _{\theta _{ \text{ old } }}\left( a_{i,t}^{m_{k}^{j}} \mid s_{i,t}^{m_{k}^{j}}\right) } \end{aligned}$$

23 $$\begin{aligned} N_{batch}= & B \cdot \sum _{k=1}^{K} \left( \left| G_{k} \right| -1 \right) \end{aligned}$$

The Advantage $A_{i}^{m_{k}^{j}}$ is calculated by Generalized Advantage Estimation (GAE), $\delta _{i,t}^{m_{k}^{j}}=r_{i,t}+\gamma V\left( s_{i,t+1}^{m_{k}^{j}}\right) -V\left( s_{i,t}^{m_{k}^{j}}\right)$. The entropy of the strategy is denoted by *S*, and a hyper parameter, $\sigma$, controls the entropy coefficient. The parameter *B* indicates the size of the batch, while *K* represents the number of groups. Furthermore, $\left| G_{k}\right|$ denotes the number of agents in group *k*. To ensure stability of the strategy iteration, policy gradient clipping is employed, where a parameter $\epsilon$ regulates the magnitude of the iteration.

Additionally, the clipped surrogate objective $\operatorname {min}\left( r_{\theta , i,t }^{m_{k}^{j} } A_{i,t}^{m_{k}^{j}},\operatorname {clip}\left( r_{\theta , i,t }^{m_{k}^{j} }, 1-\epsilon , 1+\epsilon \right) A_{i,t}^{m_{k}^{j}} \right)$ restricts overly large policy updates. This prevents destructive policy oscillations and ensures stable iterative learning, which is especially important in heterogeneous multi-agent settings. The advantage $A_{i}^{m_{k}^{j}}$ allows each member-agent to maximize actions that lead to higher expected long-term rewards while effectively reducing variance. The entropy regularization $\sigma \frac{1}{N_{batch}} \sum _{i=1}^{B} \sum _{k=1}^{K} \sum _{j=1}^{ \left| G_{k} \right| -1 } S\left[ \pi _{\theta }\left( s_{i,t}^{(m_{k}^{j})}\right) \right]$ encourages sufficient exploration during training. This is essential in our hierarchical bi-level interaction structure, where agents must explore both group-level coordination strategies and fine-grained local actions. The importance sampling ratios $r_{\theta , i,t }^{m_{k}^{j} }$ make efficient use of collected trajectories while avoiding overfitting to outdated data. The loss is computed across all agents in all groups $\sum _{k=1}^{K} \sum _{j=1}^{ \left| G_{k} \right| -1 }$ ensuring that updates account for both intra-group (isomorphic) and inter-group (heterogeneous) interactions encapsulated in our bi-level graph attention mechanism.

A value network is employed during training to reduce the variance of the policy gradient by providing an estimation of the value of the current state based on joint observable information. To stabilize critic learning and ensure consistent global learning signals, the value network is optimized using a clipped value-function loss, which approximates the expected return and mitigates fluctuations in the policy optimization process. Its optimization objective is:

24 $$\begin{aligned} L(\phi )= & \frac{1}{N_{batch}} \sum _{i=1}^{B} \sum _{k=1}^{K} \sum _{j=1}^{ \left| G_{k} \right| -1 } \nonumber \\ & \left( \operatorname { m a x } \left[ \left( V_{error}\right) ^{2}, \left( \operatorname {clip}\left( V \phi \left( s_{i,t}^{(m_{k}^{j})}\right) , V_{lowerBound},V_{upperBound}\right) -{G}_{i,t}\right) ^{2}\right] \right) . \end{aligned}$$

25 $$\begin{aligned} V_{error}= & V \phi \left( s_{i,t}^{(m_{k}^{j})}\right) -{G}_{i,t} \end{aligned}$$

26 $$\begin{aligned} V_{lowerBound}= & V \phi _{\text{ old } }\left( s_{i,t}^{(m_{k}^{j})}\right) -\epsilon \end{aligned}$$

27 $$\begin{aligned} V_{upperBound}= & V \phi _{\text{ old } }\left( s_{i,t}^{(m_{k}^{j})}\right) +\epsilon \end{aligned}$$

where ${G}_{i,t}={r}_{i,t+1}+\gamma {r}_{i,t+2}+\cdots +(\gamma )^{T-t} {r}_{i,T+1}+(\gamma )^{T+1-t} {v}({s_{i,T+1} })$ is discount reward. The algorithm of our approach can be summarized as Algorithm 1.

**Algorithm 1:**
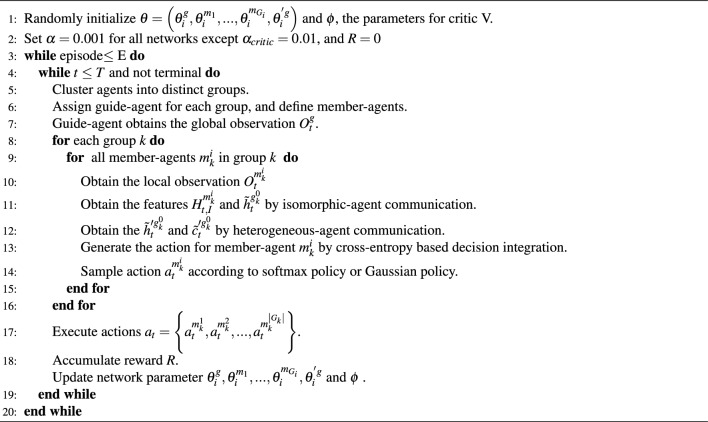
Bi-level Graph Attention Paradigm with Differential Strategy Integration for Discrete and Continuous Heterogeneous Scenarios

## Experiments

This section presents the evaluation of our algorithm in both discrete and continuous scenarios. The baselines are introduced first, followed by the exposition of the environmental settings, implement details, and results analysis for each scenario separately.

### Baselines

We compare our proposed method with six other state-of-the-art multi-agent reinforcement learning algorithms. Four of the compared algorithms use discrete action space (COMA, Reinforce+G2ANet, MS-MARL, and QMIX), while the remaining two algorithms (MADDPG and AHAC) employ continuous action space.

COMA, a CTDE-based algorithm, utilizes a centralized critic network to estimate the value function, while decentralized actors learn policies. Additionally, it employs a counterfactual baseline to address the multi-agent credit assignment.

The G2ANet uses a two-stage attention network to model inter-agent relationships. It employs a hard-attention mechanism to determine interaction between agents and a soft-attention one to learn the significance of these interactions.

MS-MARL adopts a hierarchical master-slave architecture that combines both centralized and decentralized perspectives of MARL. It facilitates strategy learning through three key aspects: composed action representation, learnable communication between agents, and independent thinking of master and slave agents.

QMIX is a value decomposition-based method that need to maintain monotonic relationship between the joint-action value and the per-agent values. A mixed network is employed to estimate joint action-values by combining the values of each agent with a non-linear weight.

MADDPG extends the single-agent reinforcement learning method to multi-agent domains with continuous action spaces using the CETD framework. To learn more robust multi-agent policies, an ensemble of policies from all agents is employed during training.

AHAC, an algorithm that incorporates the attention mechanism to represent information, is also built upon the CTDE framework. A multi-head hierarchical attention mechanism is used in critic to summarize the information from friends and enemies with different weights, which assist actors learn better strategy.

### Starcraft II

#### Environment settings

**Fig. 2:**
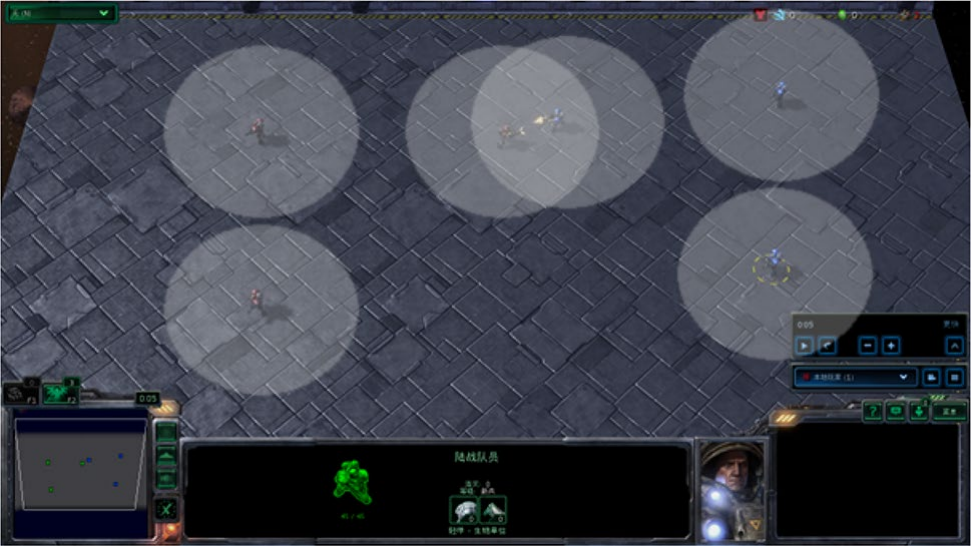
Illustrations of SMAC.

Starcraft II is a highly complex real-time strategy game, whose Micromanagement involves fine-tuned operations that are utilized to train and evaluate MARL algorithms with discrete actions. In this paper, the experiments are conducted on the StarCraft Multi-Agent Challenge (SMAC), as depicted in Fig. 2. SMAC features diverse and intricate unit attributes, leading to intricate micro-actions and interactions among agents. Each scenario entails two opposing teams, with one team controlled by a built-in AI and the other consisting of decentralized agents collaborating using tested algorithms. The winner is determined by eliminating all units of the opposing team.

**Table 1:** Detail information on the SMAC map scenarios.

Map name	The units of each side	Number of unit types	Map scale	Map classification
1c3s5z	1Colossi and 3Stalkers and 5Zealots	3	9 vs. 9	Symmetric heterogeneous
MMM	1Marines and 2Medivacs and 7Marauders	3	10 vs. 10	Symmetric heterogeneous
DMMM	2Marines and 4Medivacs and 14Marauders	3	20 vs. 20	Symmetric heterogeneous
bane_vs_bane	20Zerglings and 4banelings	2	24 vs. 24	Symmetric heterogeneous

The results are compared across multiple maps, namely 1c3s5z, MMM, bane_vs_bane, and DMMM. The first three maps exhibit both symmetry and heterogeneity, and they also contain a relatively large number of agents, making them distinctive within the original platform. To further validate the effectiveness of our algorithm, we additionally introduced the DMMM map by doubling the number of each unit in the MMM map. Detailed information about all map scenarios used in our experiments is provided in Table 1.

#### Implement details

In this paper, We follow the details of the SMAC in QMix^16^ . For clarity, the significant environment details are reiterated as follow.

**State features** Each member-agent accesses local observations from a circle with a radius of 9. The feature vector includes distance, relative x and y coordinates, unit type, and shield for each observed agent within this circle. Guide-agents, on the other hand, have global state information, including relative x and y coordinates, unit type, shield, health points, and cooldown for all agents.

**Action definition** Agents are provided with a 1x7 vector representing the available actions: no operation, stop, move in North, South, East, West directions, and target enemies for attack. These discrete actions include move [direction], attack [enemy_id], stop, and noop. The attack action [enemy_id] can only be executed if the designated enemy is within the attack range, which is constant at 6 for all agents. Additionally, only dead units perform the noop action.

**Reward definition** In the SMAC environment, agents receive a joint reward at each time step equal to the total damage inflicted on enemies. Each opponent kill rewards agents with 10 points, and an additional reward of 200 points is given upon eliminating all opponents.

**Architecture and training** For all agents, we utilize the MLP to comprehend environment observation. We employ Gated Recurrent Units (GRUs) with a 64-dimensional hidden state for member-agents and Long Short-Term Memory (LSTM) with a 128-dimensional hidden state for guide-agents to extract features. The output values from these hidden states are further processed by the MLP. Similarly, the Global Coordinator Module (GCM) uses a 128-dimensional hidden state LSTM with an MLP suffix. Value networks and Graph Attention Networks are included with single hidden layers, featuring 128 and 32 units, respectively.

**Table 2:** Main hyper-parameters of Bi-GAP for discrete scenarios.

Parameter	Value for discrete scenarios
Initial $\epsilon$, minimum $\epsilon$	0.5, 0.02
Decay factor $\gamma$	0.99
Batch size	16
Optimizer, learning rate $\alpha$	RMSprop, 1e-3
Train epochs	2e4
Evaluated cycle	100
Update epoch	5
Gradient clipped parameter	0.2
GAE $\lambda$	0.95

In the discrete scenarios, action probabilities are generated from the final layer using a bounded softmax distribution, ensuring the probability of any action is not lower than $\epsilon /|U|$. This is represented as $P(u) = (1-\epsilon )$ softmax$({\textbf {z}})_u + \epsilon /|U|$. The value of $\epsilon$ is linearly annealed from 0.5 to 0.02 over 2e4 epochs. The RMSprop optimizer is used to update the networks, with a learning rate of 1e-3 for all networks except the value network, which is set at 1e-2. The learning rate is decreased by a factor of lr_gamma every step_size epochs, with lr_gamma and step_size set to 0.9 and 500, respectively. The parameter step_mul is assigned a value of 8 to accelerate training. The algorithms are trained for 2e4 epochs, and win rates are evaluated every 100 training epochs, with each epoch consisting of 20 episodes. The networks are updated with a batch of 16 episodes, and the samples are updated every five epochs, with the policy gradient clipping parameter set to 0.2. The $\lambda$ in Generalized Advantage Estimation (GAE) used to calculate the returns is set to 0.95. The primary hyper-parameters are listed in Table 2.

#### Results and analysis

**Fig. 3:**
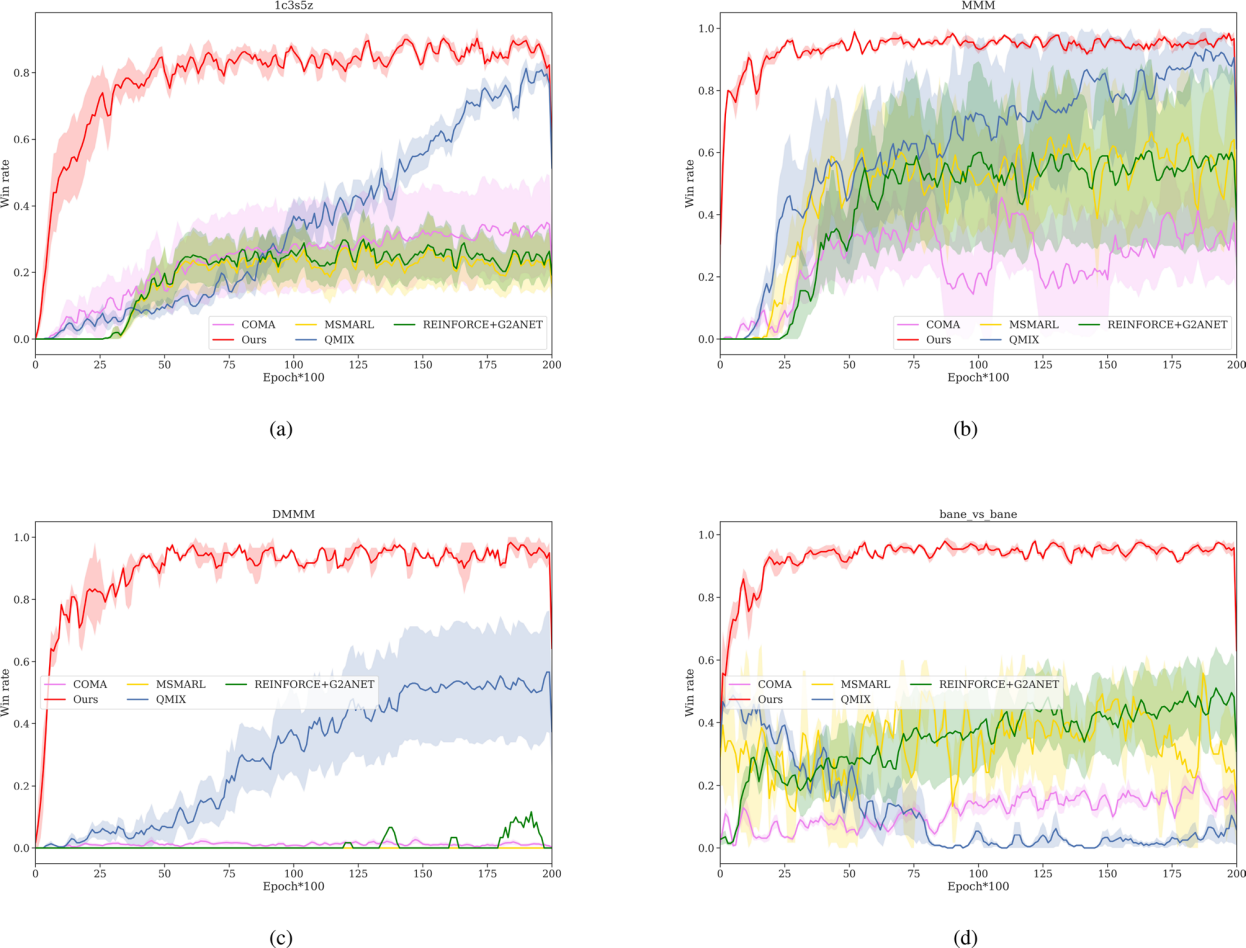
The performance of different methods on four SMAC maps: (**a**) 1c3s5z, which contains three types of 9 agents, (**b**) MMM and (**c**) DMMM involve three kinds of agents of the same type, 10 and 20 in number respectively, and (**d**) bane_vs_bane consist of two types of agents with the number of 24.

Figure 3 illustrates the performances of different algorithms, showing the average win rates on four SMAC scenarios. Specific data are also presented in Table 3 for better comprehension. The experiment was conducted five times with distinct random seeds in each scenario to enhance the reliability of the findings. Solid lines in the figures represent the average results, while shaded areas indicate the deviation of different tests.

The Bi-GAP algorithm demonstrates superior performance in heterogeneous discrete environments. Across various scenarios, as shown in Fig. 3, Bi-GAP consistently achieves win rates over 80%, outperforming all other algorithms. It exhibits significantly faster convergence speeds compared to other algorithms. In specific scenarios, such as 1c3s5z and MMM, Bi-GAP achieves win rates of 84% and 98%, respectively, with minimal fluctuation. In the DMMM scenario with exponentially increased action and state spaces, Bi-GAP still attains a high win rate of about 94%, surpassing qmix, the second-best algorithm, by 42%. In the bane_vs_bane scenario with more than 20 agents, our algorithm maintains high performance, while all other algorithms have win rates below 50%. QMIX experiences network crashes, resulting in a win rate of less than 10%. MS-MARL and REINFORCE+G2ANET exhibit similar average win rates of no more than 50% in all maps, with COMA exhibiting the lowest and most volatile win rate.

**Table 3:** The average and deviation of win rates for different methods on four SMAC maps.

Metrics	Maps	COMA	MS-MARL	QMIX	REINFORCE +G2ANET	Bi-GAP(Ours)
Win rate	1c3s5z	28.3% ± 13.4%	36.2% ± 11.8%	78.2% ± 28.3%	39.6% ± 13.5%	**84.3% ± 9.30%**
	MMM	38.4% ± 15.2%	50.1% ± 22.9%	90.6% ± 28.4%	58.1% ± 21.8%	**98.7% ± 3.6%**
	DMMM	0.2% ± 1.1%	10.8% ± 2.8%	5.3% ± 4.1%	52.1% ± 20.8%	**94.8% ± 7.8%**
	bane_vs_bane	11.9% ± 10.1%	33.6% ± 21.2%	1.7% ± 17.3%	44.9% ± 18.9%	**95.6% ± 5.2%**

Comparing the four maps, 1c3s5z exhibits the lowest winning rate despite having the fewest number of agents. The performance of an algorithm depends not only on the number of agents but also on their individual roles and characteristics. In 1c3s5z, coordinating the Protoss agents is more challenging than in the other three maps. Both Fig. 3b and c have identical agent types, but the latter has twice the number of agents. Comparing Fig. 3b,c, our algorithm’s performance only slightly decreases, with a win rate down by 5% and a convergence slowdown of around 2000 epochs. However, the other three baseline algorithms experience significant declines in win rate and convergence speed.

Figure 3a and b show that QMIX achieves comparable performance in a heterogeneous environment with fewer than 10 agents. However, for scenarios involving 20 or more agents, QMIX’s significant computational overhead from extensive value decomposition calculations makes it unsuitable. REINFORCE+G2ANET and MS-MARL are suitable for scenarios with a relatively large number of agents, but their convergence is impeded by the high variance of the policy gradient, especially with more than 20 agents. They also struggle to fully utilize agents with distinct attributes, limiting their performance. COMA, where all agents share the same networks, faces challenges in handling heterogeneous games. Moreover, as seen in Fig. 3c and d, COMA becomes increasingly susceptible to the curse of dimensionality as the number of agents grows, since its global critic must evaluate the value of each agent’s action.

### Predator-prey in multi-agent particle environment

#### Environment settings

**Fig. 4:**
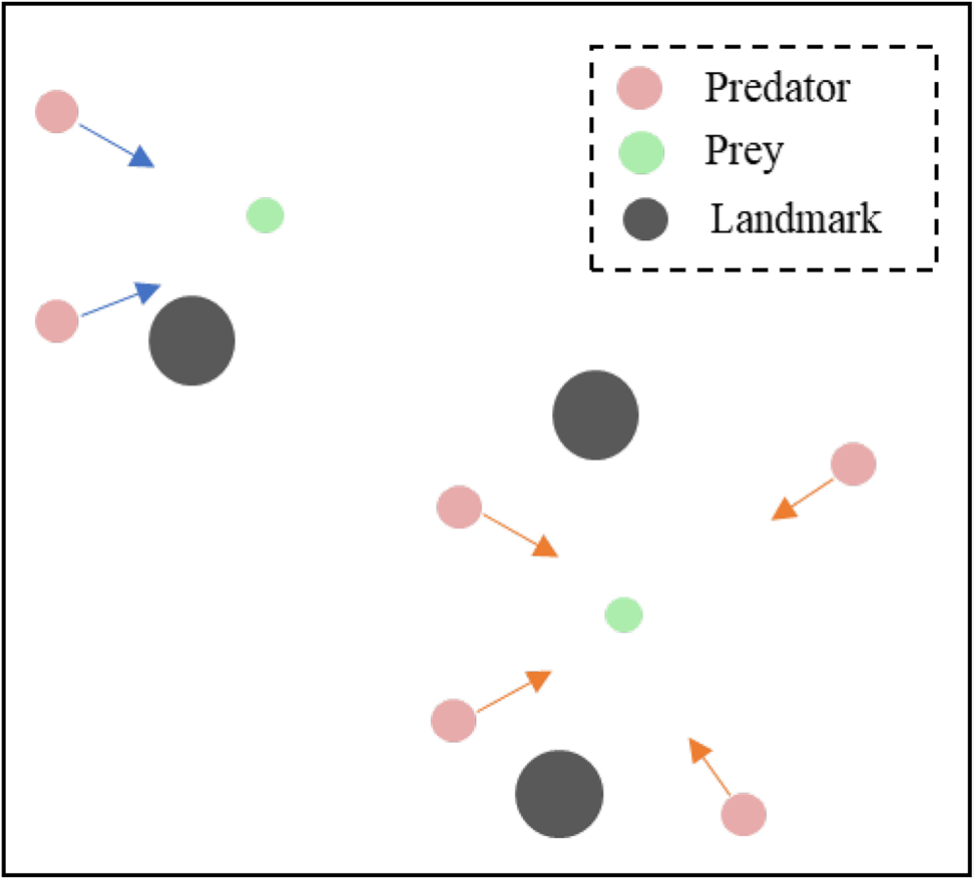
Illustrations of Predator-Prey.

As illustrated in Fig. 4, Predator-Prey, a mixed cooperative-competition game, often used for testing MARL algorithm with continuous action. It involves not only agents categorized as predators (red) and prey (green), but also landmarks that the agents must navigate around. Of the agents, the predators outnumber the prey, necessitating that they be assigned a slower speed in order to balance the relative abilities of the two sides. And we train the preys by the DDPG.

**Table 4:** Detailed information on the Predator-Prey scenarios.

Scenarios	Predators		Preys		Number of Landmarks	Viewpoint
	Numbers	Acceleration	Numbers	Acceleration		
6 vs. 2	2	2.5	2	4.0	3	2x2
	4	3.0				
9 vs. 3	2	2.5	3	4.0	3	2x2
	4	3.0				
	3	3.5				
6 vs. 2 (Partial Observation)	2	2.5	2	4.0	3	1x1
	4	3.0				
9 vs. 3 (Partial Observation)	2	2.5	3	4.0	3	1x1
	4	3.0				
	3	3.5				

We evaluate the algorithms in the 6 vs. 2 and 9 vs. 3 games, introducing variations in predator accelerations to create heterogeneous environments. To assess the effectiveness of our communication model, predators have local viewpoints, allowing them to observe within their field of view. The agents initially move within a 2 by 2 units rectangular area. In the local observation setting, the predators’ observation range is limited to a 1 by 1 units rectangular area centered on their position, contrasting with full observation. Further details on the Predator-Prey scenarios can be found in Table 4.

#### Implement details

The experiments in this part employ the Predator-Prey settings as proposed in MADDPG^9^. To provide lucidity, we recapitulate the crucial environment specifics as presented below.

**State Features** Predators and prey are characterized by feature vectors that encompass various attributes, such as their respective speeds, positions, the relative positions of all landmarks, the relative positions of other agents, and the speeds of their counterparts (preys for predators and other preys for preys).

**Action Definition** Actions are represented by a one-dimensional vector of size 1x5. The first element corresponds to no operation, while the subsequent elements represent acceleration in the directions of North, South, East, and West. The agents obtain the velocity and position for the next time step from this vector.

**Reward Definition** Similar to the MADDPG setting, the predators are rewarded with a positive reward of +10 when they successfully capture the preys.

**Architecture and Training** In continuous scenarios, the network architectures are similar to their discrete counterparts, with most parameters following the specifications outlined earlier. However, a small subset of parameters requires reconfiguration. Specifically, the action is sampled from a Gaussian policy with a fixed variance of $\sigma =0.05$. The ADAM optimizer is used, and all network learning rates are initialized to 1e-2. Due to sparser rewards in Predator-Prey compared to Starcraft II, more epochs (3e4) are required for effective training. To enhance policy learning efficiency and prevent excessive data reuse, the update epoch is reduced from 5 to 1. The primary hyper-parameters are listed in Table 5.

**Table 5:** Main hyper-parameters of Bi-GAP for continuous scenarios.

Parameter	Value for continuous scenarios
Variance of Gaussian policy $\sigma$	0.05
Decay factor $\gamma$	0.99
Batch size	8
Optimizer, learning rate $\alpha$	ADAM, 1e-2
Train epochs	3e4
Evaluated cycle	100
Update epoch	1
Gradient clipped parameter	0.2
GAE $\lambda$	0.95

#### Results and analysis

**Fig. 5:**
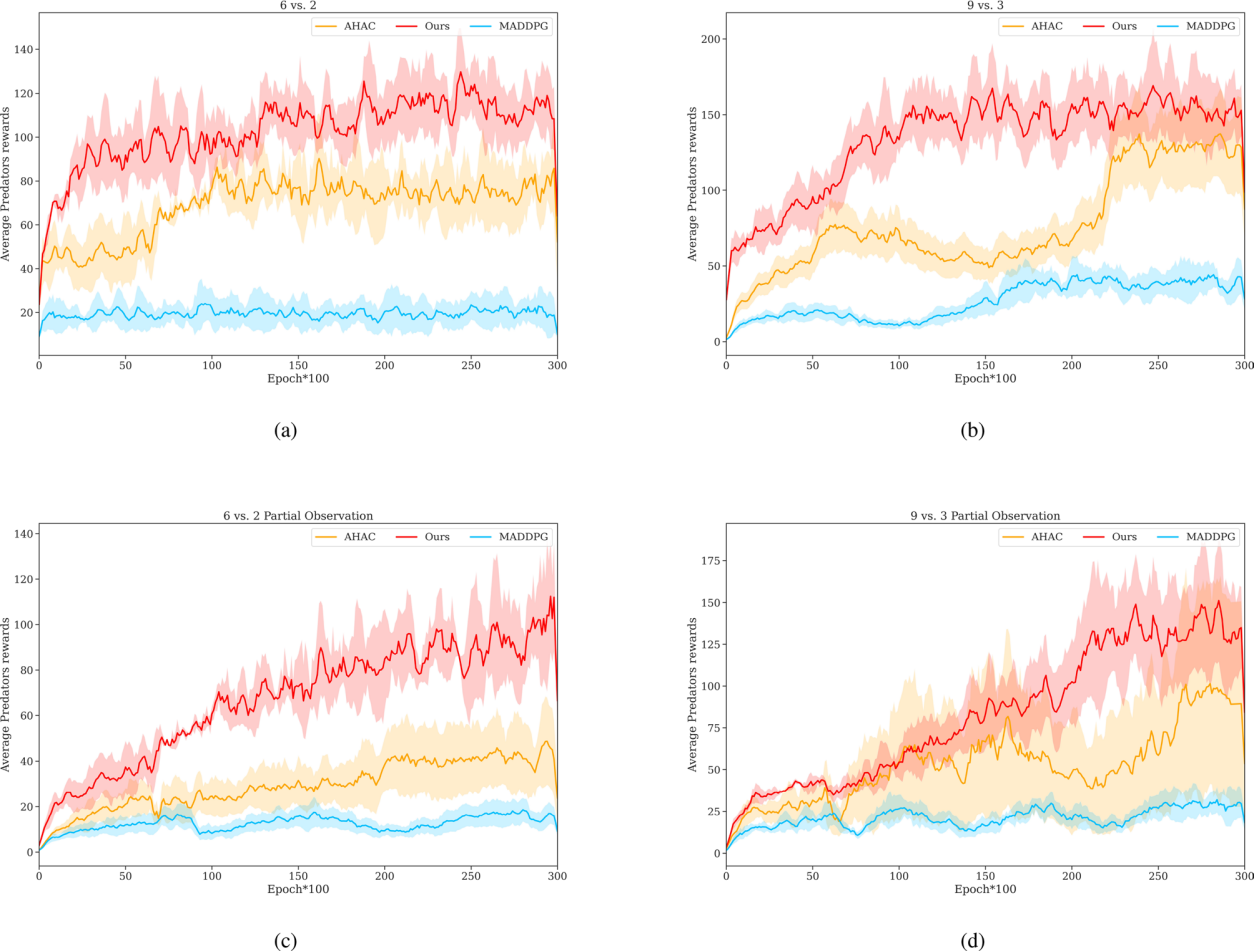
The performance of different methods on four Predator-Prey games: (**a**) 6 vs. 2 has 6 Predators with 2 types of accelerations and 2 preys, (**b**) 9 vs. 3 includes 9 Predators with 3 kinds of accelerations and 3 preys, (**c**) 6 vs. 2. Partial Observation and (**d**) 9 vs. 3. Partial Observation differ from (**a**) and (**b**) in equipping predators with local viewpoints.

Figure 5 visually represents the performance of various algorithms, showing the average rewards obtained in four different Predator-Prey scenarios. Specific data are presented in Table 6 for clarity. Similar to the discrete scenarios, each experiment was conducted five times with different random seeds. Mean results and variance are indicated by solid lines and shaded regions, respectively.

Our Bi-GAP algorithm demonstrates competitive performance in heterogeneous continuous environments, as shown in Fig. 5. In the 6 vs. 2 game, Bi-GAP achieves a mean predator reward of 102, outperforming AHAC by 34 and MADDPG by 83. It exhibits faster convergence, reaching the first phase convergence at the 2500th epoch and the final convergence at the 14000th epoch with a reward of 110. In contrast, AHAC converges to around 75 at the 10000th epoch, while MADDPG converges quickly but obtains a significantly lower reward of 20 due to the critic’s inability to discriminate agents with different attributes and the challenges posed by the massive exception curse. In Fig. 5b, with an increase in the number of predators and prey in the 9 vs. 3 game, our Bi-GAP achieves higher rewards, surpassing AHAC and MADDPG by 59 and 109, respectively, with an increase of 136. The larger agent population provides more opportunities for predator-prey interactions, leading to increased rewards for all three algorithms. However, the increased complexity and diversity of information interaction and optimal strategies result in a slowdown in convergence speed for all three algorithms. Bi-GAP experiences a slight slowdown, reaching convergence at around the 10000th epoch, while AHAC encounters further turbulence during convergence due to the challenges posed by heterogeneous agents.

**Table 6:** The average and standard deviation of predators rewards for different methods on four predator-prey games.

Metrics	Scenarios	MADDPG	AHAC	Bi-GAP(Ours)
Predators Rewards	6 vs. 2	19.5 ± 1.2	68.7 ± 7.6	**102.2** ± **6.2**
	9 vs. 3	27.1 ± 4.9	77.2 ± 12.9	**136.8** ± **9.6**
	6 vs. 2(Partial Observation)	14.7 ± 2.1	29.1 ± 6.3	**66.8** ± **7.3**
	9 vs. 3(Partial Observation)	21.1 ± 2.7	52.7 ± 18.6	**83.2** ± **15.4**

The limited viewpoint poses challenges for learning the global optimal strategy, making effective information interaction and strategy optimization crucial. To further validate our algorithm’s superiority, we evaluate algorithms in partially observable heterogeneous Predator-Prey games. The results in Fig. 5c and d show decreased rewards for all tested algorithms, especially the baselines. In the 6 vs. 2 (Partial Observation) game, our Bi-GAP achieves a reward of 66 for the predators, which is 36 lower than in the 6 vs. 2 fully observable game but still 37 higher than the second-best AHAC. Despite partial observation leading to strategy learning instability, Bi-GAP demonstrates a relatively stable upward reward trend, albeit at a slower pace than fully observable settings. This can be attributed to effective Bi-level communication and reasonable decision integration technique. In contrast, AHAC and MADDPG exhibit more pronounced fluctuations, particularly evident in Fig. 5d. Comparing performance between partial and fully observed environments, the gap in converged reward values between Bi-GAP and the baselines is larger in the partially observed settings, further emphasizing our algorithm’s superior performance.

## Conclusion and future work

Efficient communication and effective strategy optimization are crucial for addressing challenging heterogeneous multi-agent games with discrete or continuous action spaces, particularly as the number of agents increases. In this paper, a Bi-level Graph Attention Network is leveraged to dynamically establish effective communication channels among isomorphic agents and heterogeneous groups. Moreover, member-agents intelligently incorporate policy guidance from an exclusive guide-agent to optimize their actions. These mechanisms effectively mitigate the impact of incomplete information and decision-making errors, enhancing the performance of heterogeneous MARL in complex environments. The proposed approach opens new avenues for multi-agent policy learning as agent numbers grow and offers insights for future research on solving practical tasks.

In the current work, agents make decisions based primarily on peer information and type-based groupings. While our framework is conceptually compatible with dynamic, mixed-type grouping—where heterogeneous agents could be grouped by factors such as spatial proximity, tactical roles, or task objectives—such scenarios have not been empirically evaluated in this study and are left as an important direction for future work. Additionally, future investigations will consider incorporating opponent information, handling intermittent or missing observations, and exploring how the framework can maintain efficient communication, effective coordination, and robust learning as agent numbers and heterogeneity continue to increase.

## Data Availability

The datasets generated and/or analyzed during the current study are not publicly available due to proprietary reasons but are available from the corresponding author on reasonable request.
